# Prediction of RNA-binding amino acids from protein and RNA sequences

**DOI:** 10.1186/1471-2105-12-S13-S7

**Published:** 2011-11-30

**Authors:** Sungwook Choi, Kyungsook Han

**Affiliations:** 1 School of Computer Science and Engineering, Inha University, Inchon 402-751, South Korea

## Abstract

**Background:**

Many learning approaches to predicting RNA-binding residues in a protein sequence construct a non-redundant training dataset based on the sequence similarity. The sequence similarity-based method either takes a whole sequence or discards it for a training dataset. However, similar sequences or even identical sequences can have different interaction sites depending on their interaction partners, and this information is lost when the sequences are removed. Furthermore, a training dataset constructed by the sequence similarity-based method may contain redundant data when the remaining sequence contains similar subsequences within the sequence. In addition to the problem with the training dataset, most approaches do not consider the interacting partner (i.e., RNA) of a protein when they predict RNA-binding amino acids. Thus, they always predict the same RNA-binding sites for a given protein sequence even if the protein binds to different RNA molecules.

**Results:**

We developed a feature vector-based method that removes data redundancy for a non-redundant training dataset. The feature vector-based method constructed a larger training dataset than the standard sequence similarity-based method, yet the dataset contained no redundant data. We identified effective features of protein and RNA (the interaction propensity of amino acid triplets, global features of the protein sequence, and RNA feature) for predicting RNA-binding residues. Using the method and features, we built a support vector machine (SVM) model that predicted RNA-binding residues in a protein sequence. Our SVM model showed an accuracy of 84.2%, an F-measure of 76.1%, and a correlation coefficient of 0.41 with 5-fold cross validation on a non-redundant dataset from 3,149 protein-RNA interacting pairs. In an independent test dataset that does not include the 3,149 pairs and were not used in training the SVM model, it achieved an accuracy of 90.3%, an F-measure of 72.8%, and a correlation coefficient of 0.24. Comparison with other methods on the same datasets demonstrated that our model was better than the others.

**Conclusions:**

The feature vector-based redundancy reduction method is powerful for constructing a non-redundant training dataset for a learning model since it generates a larger dataset with non-redundant data than the standard sequence similarity-based method. Including the features of both RNA and protein sequences in a feature vector results in better performance than using the protein features only when predicting the RNA-binding residues in a protein sequence.

## Background

Interactions between proteins and RNA are fundamental to many cellular processes [1]. Much experimental and theoretical effort has been made to study protein-RNA interactions, but their precise mechanism is not fully understood. Motivated by the recent increase in structures of protein-RNA complexes, several theoretical studies such as supervised learning have been carried out to predict RNA-binding residues in protein sequences. For example, BindN [2] uses a support vector machine (SVM) to predict the RNA- or DNA-binding residues in a protein sequence based on the chemical properties of amino acids. RNABindR [3,4] predicts the RNA-binding residues in a protein sequence using a Naïve Bayes classifier. However, none of these consider interacting partners (i.e., RNA) for a given protein when predicting RNA-binding amino acids. Thus, they always predict the same RNA-binding sites for a given protein sequence even if the protein binds to different RNA molecules.

We previously studied the interactions between protein and RNA [5,6]. In an effort to discover binding-specific features of amino acids and nucleotides, we performed an extensive analysis of the recent structures of protein-RNA complexes and computed several types of interaction propensity (IP) between amino acids and nucleotides [7]. Our analysis revealed that the IP of amino acid triplets has a higher binding specificity than IP of individual amino acids or other biochemical properties. In this study, we modified the previous interaction propensity of amino acid triplets and computed the new interaction propensity from more structures of protein-RNA complexes. In addition to the interaction propensity, we identified several features of protein and RNA sequences which are effective for predicting RNA-binding amino acids in a protein sequence.

**Figure 1:**
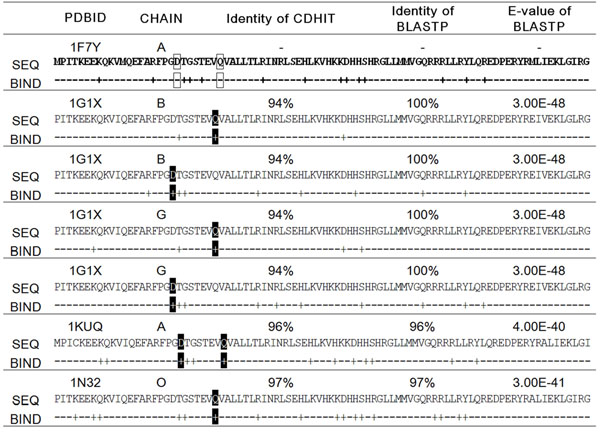
**The binding information loss during the redundancy reduction based on measuring sequence similarity**. The clustered protein sequences by CDHIT. Protein chain A of the protein-RNA complex 1F7Y (1F7Y:A) was selected as the representative sequence because it was the longest. In the representative sequence, the boxed residues were determined as non-binding residues, but those residues in similar locations were determined to be binding residues in the non-selected protein sequences. Hence, the binding information of non-selected protein sequences was not contained in input training data which would only include the binding information of selected sequences.

In supervised learning approaches, preparing enough training data is crucial for its success, but the training data should be non-redundant and representative. Many learning approaches to predicting RNA-binding residues construct a training dataset based on the similarity of protein sequences without considering their binding and partial sequence information [2-4,8-10]. However, these approaches eliminate much binding information from a training dataset, which would otherwise be valuable for predicting binding sites. Consider the protein sequences of Figure 1, which were grouped by CD-HIT [11] based on sequence similarity. CD-HIT selects the protein chain A of the protein-RNA complex 1F7Y (1F7Y:A) as the representative sequence of the cluster since it is the longest sequence in the cluster. For a small training dataset, removing all the sequences except the representative one will make the training dataset smaller beyond the boundary of practicality. More importantly, the binding information of the removed sequences is lost during the redundancy reduction of the sequences. Including all the redundant sequences of the cluster in a training dataset would yield a classifier prone to over-fitting due to exposure to a highly redundant training dataset.

In the standard redundancy reduction practice for sequence data, a sequence is included in a training dataset as a whole or discarded from a training dataset. It is a take all or nothing approach, so no partial sequence of the original sequence is allowed to be included in a training dataset. As a solution, we proposed a feature vector-based reduction of data redundancy. In the feature vector-based approach, two identical feature vectors with different binding labels (i.e., one is binding and the other is non-binding) are considered as different feature vectors and both are included in a training dataset. The feature vector-based redundancy reduction method constructs a powerful training dataset, especially when there is not sufficient data available.

This paper describes a support vector machine (SVM) model that predicts RNA-binding residues by considering both RNA and protein sequences. The SVM model was trained on a non-redundant dataset constructed by the feature vector-based redundancy reduction method. To the best of our knowledge, this is the first attempt to predict RNA-binding amino acids by considering the RNA sequence that interacts with the protein. The rest of the paper presents the details of the SVM model and its experimental results.

## Methods

### Definition of protein-RNA binding sites

Different studies have used slightly different criteria for protein-RNA binding sites. For example, in BindN [2] and RNABindR [3,4] an amino acid with an atom within a distance of 5Å from any other atom of a nucleotide was considered to be an RNA-binding amino acid. However, we use stricter criteria than that. In our study, an RNA-binding amino acid should satisfy the following geometric criteria for hydrogen boding (H bond) interaction with RNA: the contacts with the donor-acceptor (D-A) distance < 3.9*Å*, the hydrogen-acceptor (H-A) distance < 2.5*Å*, the donor-hydrogen-acceptor (D-H-A) angle > 90° and the hydrogen-acceptor-acceptor antecedent (H-A-AA) angle > 90°.

These criteria are slightly different from the ones used in our previous studies [6,12] but they are the most commonly used criteria for H bonds. In particular, the criteria of a H-A distance < 2.5*Å* and a D-H-A angle > 90° are essential for the H bonds [13].

### Interaction propensity of amino acid triplets

A same amino acid can have different interaction propensities with different neighbors or at different secondary structures. We computed the interaction propensity of three consecutive amino acids in a sequence (called amino acid triplet or triple amino acids) and used the interaction propensity to predict RNA-binding residues. The interaction propensity *IP_tb_* between the amino acid triplet *t* and the nucleotide *b* is defined by equation (1).(1)

This definition of *IP_tb_* in equation (1) slightly differs from the definition we used in our previous studies [5-7]: (1) *IP_tb_* uses the inverse of the value that the H-A distance multiplied by cosine of D-A-H angle of the H-bonds between an amino acid in a triplet and a nucleotide instead of using the H-bonds between an amino acid and a nucleotide, and (2) *IP_tb_* uses amino acid triplets instead of individual amino acids.

In equation (1),  is the sum of the inverse projected distance of H-A on the D-A between triplet *t* and the binding nucleotide *b*, *N_PR_* is the total number of amino acid triplets that bind to any nucleotides, *N_t_* is the number of triplets *t*, *N_P_* is the total number of amino acid triplets, *N_b_* is the number of nucleotide *b* and *N_R_* is the total number of nucleotides in the dataset. The purpose of using the projected distance of H-A distance on D-A in the H bonds is to consider H-A distance as well as the locational relationship to D-A. The purpose of using the inverse value is to assign higher IP values to the H-bonds with the close donor-acceptor pairs than those with the distant donor-acceptor pairs. Since there are 20^3^ = 8,000 amino acid triplets and 4 nucleotides, we computed 32,000 IPs between amino acid triplets and nucleotides.

### Encoding a feature vector

To predict RNA-binding amino acids in the protein sequence, we represented several features of protein and RNA sequences in a feature vector. The features can be categorized into three different feature types: (1) global features of the protein sequence, (2) local features of amino acids, and (3) partner features. The global features represent the entire sequence information of the target residue when the local features represent the individual information of the residue. Since our prediction model is to predict different binding sites in a protein sequence depending on a partner sequence, the information of an interacting partner sequence was used with the other features.

**Figure 2:**
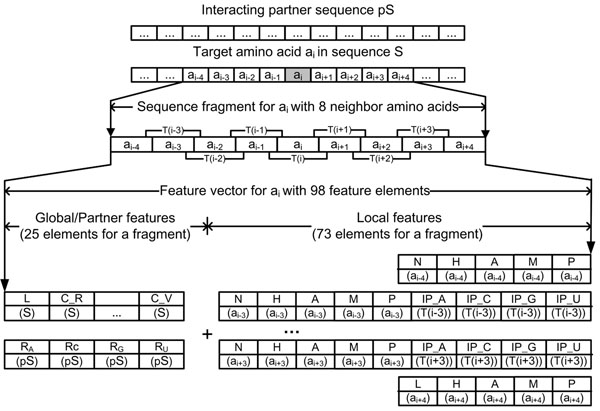
**The structure of a feature vector with the window of 9 amino acids**. A window of 9 amino acids corresponds to 7 overlapping triplets: *T*(*i* – 3), *T*(*i* – 2), …, *T*(*i* + 2), *T*(*i* + 3). 21 global feature elements (1 L and 20 Cs) and 4 RNA feature elements (R*_A_*, R*_C_*, R*_G_*, R*_U_*) are encoded once for a given pair of protein and RNA sequences. 9 local feature elements (N, H, A, M, P and 4 IPs) are encoded for 7 internal residues, and 5 local feature elements (N, H, A, M, P) for 2 terminal residues. Thus, the feature vector representing a window of 9 residues has a total of 98 (=21+4+9×7+5×2) feature elements.

• **Global features of the protein sequence included the sequence** length (L) and amino acid composition (C). The amino acid composition represented the frequencies of 20 amino acids in a protein sequence. The global features required a total of 21 elements in a feature vector, one for the sequence length and 20 for the amino acid composition.

• **Local features of amino acids** included the normalized position (N), hydropathy (H), accessible surface area (A), molecular mass (M), and side chain pK*_a_* (P) value of an amino acid, the interaction propensity (IP) of an amino acid triplet. IP is represented as 4 elements, IP_A, IP_C, IP_G, and IP_U, in which IP_A denotes the interaction propensity of the amino acid triplet with the nucleotide adenine (A) (Figure 2). The normalized position of an amino acid in the sequence is calculated by equation (2). Except for the normalized position, a same amino acid or amino acid triplet has the same value for the local features.(2)

• **Partner features** represent the feature of the RNA (R) sequence that interacts with the protein. For each of the four nucleotides, we encoded the sum of the normalized position of the nucleotide in the RNA sequence. This feature is computed by equation (3) and represented as 4 elements (R*_A_*, R*_C_*, R*_G_*, R*_U_*) in a feature vector. Due to these elements, identical amino acid sequences can be encoded into different feature vectors if they interact with different RNA sequences.(3)

Each of the feature elements is normalized into a value in the range of [0, 1] when it is represented in a feature vector. The global features of a protein (1 element for L and 20 elements for C) and its partner feature (4 elements for R) are represented once for the entire protein sequence, but the local features of a protein should be represented for each internal residue (5 elements for N, H, A, M, and P and 4 elements for IP). The IP is not defined for the terminal residue of a window (e.g., *a_i_*_–4_ and *a_i_*_+4_ in Figure 2), so only 5 elements are represented for the terminal residues.

Since we use overlapping triplets for encoding a sequence, a sliding window of *w* residues corresponds to *w* – 2 triplets. When a sliding window of *w* residues is used, the feature vector for residue *i* starts with residue *i* - (*w* – 1)/2 and covers the triplets *T*(*i* – (*w* – 1)/2 – 1),*T*(*i* – (*w* – 3)/2 – 1), …,*T*(*i* + (*w* – 3)/2 – 1) and *T*(*i* + (*w* – 1)/2 – 1). Thus, a sequence fragment of *w* residues is encoded as a feature vector of 9w+17 elements: 21 global elements (1 L and 20 Cs), 4 RNA elements (R*_A_*, R*_C_*, R*_G_* and R*_U_*), 9 local elements (N, H, A, M, P and 4 IPs) for *w* – 2 internal residues, and 5 local elements (N, H, A, M and P) for 2 terminal residues. A feature vector is labeled +1 (positive) if the middle residue of the sequence fragment is a binding residue, and -1 (negative) otherwise. Figure 2 shows an example of a feature vector for an amino acid sequence with a window of 9 amino acids.

### Feature vector-based reduction of data redundancy

All of the protein sequences in the protein-RNA interacting pairs are segmented into overlapping sequence fragments of a window size *w*. From a protein sequence of *n* amino acids, *n* sequence fragments are generated and each sequence fragment is encoded into a feature vector. Feature vectors are considered identical only when they have the same elements and labels. When a prediction model is trained by redundant data, a bias towards the over-represented data is introduced during prediction. Thus, a training dataset should be constructed with the most representative data after removing redundant data.

**Figure 3:**
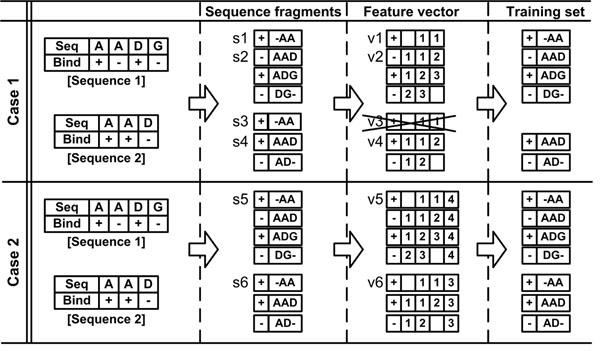
**An example of feature vector-based reduction of data redundancy**. Case 1 shows the procedure of the feature vector-based redundancy reduction method with a hypothetical amino acid-based feature when there is a different typed feature: sequence length feature is combined with the amino acid-based feature to encode a feature vector in case 2. The sequence fragment (s3) is not included in the training dataset in case 1 because it generates a redundant feature vector with the one from the sequence fragment (s1). However, the sequence fragments (s5, s6) become the non-identical feature vectors (v5, v6) when using the sequence length feature in case 2 and both sequence fragments are included in the training dataset.

We removed redundant data based on the feature vector representing the data. Figure 3 explains our method with hypothetical sequences and features. In case 1 of Figure 3, the sequence fragments s1 and s3 have the same amino acid sequence and the middle amino acids of s1 and s3 are both binding sites. Thus, the feature vectors v1 and v3, representing the sequence fragments s1 and s3, have the same vector elements and the label. To remove redundant data in a training dataset, only one sequence fragment (s1 in this example) is left and s3 is discarded. On the other hand, the sequence fragments s2 and s4 have the same vector elements but different labels, so their feature vectors v2 and v4 are not identical. Both s2 and s4 are included in the training dataset. In case 2 of Figure 3, an additional feature of the protein, sequence length, is included in a feature vector. Then, the feature vectors v5 and v6 representing the sequence fragments s5 and s6 are no longer the same.

**Figure 4:**
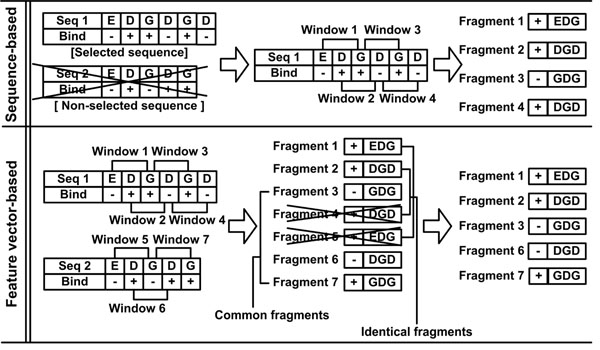
**Comparison of the sequence similarity-based method and the feature vector-based method for reducing data redundancy**. The sequence similarity-based method removes an entire sequence that is identical or similar to other sequences. When similar sequences are eliminated from a dataset, their binding information is also lost. When the remaining sequence contains repetitive subsequences, redundant data are generated from the subsequences. The feature vector-based method first represents every possible subsequence and its binding information as a feature vector. A subsequence is removed only when it has the same feature vector as others. Subsequences with the same amino acid sequence but different binding information are considered different and both are kept in the training dataset.

Figure 4 compares the feature vector-based redundancy reduction method with the standard redundancy reduction method, which reduces data redundancy based on the sequence similarity. The feature vector-based method constructs a non-redundant training dataset with all possible sequence fragments in the protein sequences, but the sequence similarity-based method discards some sequence fragments and constructs a smaller training dataset than the feature vector-based method. It is also noticeable that the sequence similarity-based method kept the redundant data (Fragment 2 and Fragment 4) in the training dataset, whereas the feature vector-based method did not include redundant data in the training dataset by considering the feature vectors and their labels. When a prediction model is trained by redundant data, the model is biased toward the over-represented data.

### Performance measures

The performance of the prediction model was evaluated using seven different measures: sensitivity (Sn), specificity (Sp), precision rate (Pr), accuracy (Ac), net prediction (NP), F-measure (Fm) and correlation coefficient (CC).(4)(5)(6)(7)(8)(9)(10)

In these equations, the true positives (TP) are binding residues that are predicted as the binding residues, the true negatives (TN) are non-binding residues that are predicted as the non-binding residues, the false positives (FP) are non-binding residues that are predicted as the binding residues, and the false negatives (FN) are binding residues that are predicted as the non-binding residues.

Sensitivity is the percentage of amino acids that are RNA-binding and are correctly predicted as RNA-binding. Specificity is the percentage of amino acids that are not RNA-binding and are correctly predicted as non-binding. Accuracy is the percentage of amino acids that are correctly predicted. But, accuracy may be misleading in highly imbalanced datasets. For example, in a dataset of 10 positive and 90 negative samples, the accuracy becomes as high as 90% if all the samples are classified as negative. Net prediction is the average of sensitivity and specificity. The correlation coefficient is the best single measure for comparing the overall performance of different methods [14].

## Results and discussion

### Datasets of protein-RNA interactions

We constructed three different protein-RNA interaction datasets: PRI3149, PRI727 and PRI267. For the PRI3149 dataset, the protein-RNA complexes were obtained from the Protein Data Bank (PDB) [15]. As of November 2009, there were 442 protein-RNA complexes that were determined by X-ray crystallography with a resolution of 3.0*Å* or better. After applying the geometric criteria for H bonds to 442 protein-RNA complexes, 429 protein-RNA complexes containing 3,149 pairs of interacting protein-RNA sequences were left that satisfied the criteria. If a protein *p* interacted with two different RNAs *r*1 and *r*2, both pairs *p* – *r*1 and *p* – *r*2 were included in the dataset. The 3,149 protein-RNA interacting pairs were formed by 2,663 protein sequences and 812 RNA sequences. From the PRI3149 dataset, we constructed a set of non-redundant feature vectors to train the SVM model.

The PRI727 and PRI267 datasets were constructed independently from the PRI3149 dataset solely for testing different methods of predicting RNA-binding residues in the protein sequence. We obtained a total of 107 protein-RNA complexes that had been deposited in PDB since November 2009. After applying the geometric criteria for H bonds to the 107 protein-RNA complexes, 727 protein-RNA interacting pairs with 592 protein sequences and 244 RNA sequences were left to form the PRI727 dataset.

For a more rigorous evaluation, any pair of protein and RNA sequences in the PRI727 dataset with >60% sequence identity to the sequences in the PRI3149 was removed. As a result, 267 protein-RNA interacting pairs with 192 protein sequences and 211 RNA sequences were left to form the PRI267 dataset. Details of the datasets are available as Additional Files 1, 2, 3.

### Feature vector-based reduction of data redundancy

The PRI3149 dataset of 3,149 protein-RNA interacting pairs initially contains 59,398 RNA-binding residues and 542,627 non-binding residues. If redundant data is not removed, the number of positive sequence fragments is the same as that of binding residues and the number of negative sequence fragments is the same as that of non-binding residues. We represented the 3,149 protein-RNA interacting pairs as feature vectors using two different combinations (all protein features and RNA features vs. local features of protein) of their features and applied the feature vector-based redundancy reduction method to the feature vectors.

Table 1 shows the number of remaining feature vectors after applying the feature vector-based redundancy reduction method to the PRI3149 dataset. Common vectors in Table 1 denote the feature vectors with the same vector elements but with different binding labels (’+1’ for binding and ’-1’ for non-binding) (Figure 4). It is harder to separate different classes in the data with more common feature vectors than those with fewer common feature vectors. As shown in Table 1, using all the features (protein sequence length, amino acid composition, normalized position, hydropathy, accessible surface area, molecular mass, and side chain *pK_a_* of an amino acid, IP of an amino acid triplet, sum of the normalized position of each nucleotide type) produced more feature vectors but a smaller proportion of common feature vectors than using the 6 local features of protein (normalized position, hydropathy, accessible surface area, molecular mass, and side chain *pK_a_* of an amino acid, IP of an amino acid triplet) consistently in all window sizes. When the 6 local features of sequence fragments were represented, the feature vector-based redundancy reduction method with a larger window size constructed a larger non-redundant dataset. However, when the 9 features were represented, the feature vector-based redundancy reduction method constructed non-redundant datasets of similar size irrespective of the window size.

**Table 1:** Feature vectors generated by the feature vector-based redundancy reduction method to the PRI3149 dataset.

Window size	#Positive feature vectors	#Negative feature vectors	#Total vectors	#Common vectors
with 9 features (L, C, N, H, A, M, *pK_a_*, IP, and R)				

1	21,282	198,578	219,860	2,811
3	21,282	198,585	219,867	2,811
5	21,283	198,590	219,873	2,811
7	21,283	198,596	219,879	2,811
9	21,284	198,601	219,885	2,811
11	21,284	198,606	219,890	2,811
13	21,284	198,611	219,895	2,811
15	21,284	198,616	219,900	2,811

with 6 features (N, H, A, M, *pK_a_*, and IP)				

1	6,286	74,829	81,115	3,641
3	6,618	81,390	88,008	3,164
5	6,658	81,729	88,387	3,164
7	6,681	81,891	88,572	3,168
9	6,702	82,010	88,712	3,170
11	6,710	82,129	88,839	3,168
13	6,720	82,242	88,962	3,169
15	6,733	82,349	89,082	3,173

The number of non-redundant feature vectors generated from the PRI3149 dataset by the feature vector-based redundancy reduction method with various window sizes. Common vectors denote the feature vectors with the same vector elements but with different classes. 9 features: protein sequence length (L), amino acid composition (C), normalized position (N), hydropathy (H), accessible surface area (A), molecular mass (M), and side chain *pK_a_* of an amino acid, IP of an amino acid triplet (IP), sum of the snormalized position of each nucleotide type (R). 6 features: N, H, A, M, *pK_a_*, and IP of an amino acid.

Table 2 compares the performance of the feature vector-based redundancy reduction method with that of the sequence similarity-based redundancy reduction method in the PRI727 and PRI267 datasets. S-method is the sequence similarity-based redundancy reduction using the CD-HIT program. The number in the parenthesis represents the sequence identity threshold of CD-HIT clusters, and the longest sequence of each cluster was included in a training dataset. F-method is the feature vector-based redundancy reduction. The only difference between S-method and F-method was in their training datasets. The SVM model was trained and tested using 9 features and a window size of 15. In both the PRI727 and PRI267 datasets, F-method was better than S-method in all performance measures.

**Table 2:** Comparison of the redundancy reduction methods for training datasets.

Dataset construction	Sensitivity (%)	Specificity (%)	Accuracy (%)	NP (%)	Fm (%)	CC
PRI727 dataset						

S-method (100%)	84.1	75.8	76.3	80.0	79.7	0.32
S-method (80%)	84.9	74.3	74.9	79.6	79.2	0.31
S-method (60%)	85.4	72.7	73.5	79.1	78.6	0.30
F-method	87.2	81.7	82.1	84.5	84.4	0.40

PRI267 dataset						

S-method (100%)	46.4	86.8	85.9	66.6	60.5	0.14
S-method (80%)	48.4	85.7	84.9	67.2	62.2	0.14
S-method (60%)	49.6	84.5	83.8	67.0	62.5	0.13
F-method	60.7	91.0	90.3	75.8	72.8	0.24

S-method is the sequence similarity-based redundancy reduction using the CD-HIT program. The number in the parenthesis indicates the sequence identity threshold of CD-HIT clusters. F-method is the feature vector-based redundancy reduction. The SVM model was trained and tested using 9 features and a window size of 15. NP: net prediction. Fm: F-measure. CC: correlation coefficient.

### Interaction propensity of amino acid triplets with RNA

By equation (1), we computed 32,000 interaction propensities between the 8,000 amino acid triplets and 4 nucleotides with the PRI3149 dataset. 18,301 IPs (57.2%) out of the total 32,000 IPs have non-zero values, ranging from 0.0 to 4.789796. The pair of (SHK, U) had the highest IP value (4.79) and CRR showed high IP values (2.46) with all nucleotides on average. In contrast, 2,238 IPs had zero values with all nucleotides.

In addition to the IP of amino acid triplets, we computed the four RNA feature elements (R*_A_*, R*_C_*, R*_G_*, R*_U_*) for the RNA sequences in the PRI3149 dataset using equation (3). The PRI3149 dataset contains 812 RNA sequences, and only 312 sequences are distinguishable from each other. When we represented the four RNA features for the 312 sequences, they became unique feature vectors. The interaction propensities of amino acid triplets and the RNA feature elements computed for the PRI3149 dataset are available Additional Files 4 and 5.

To examine the effect of several definitions of the interaction propensity of amino acids with RNA on prediction performance, we encoded the non-redundant dataset using 3 different definitions of IP: the interaction propensity sIP of single amino acids [5,6], the interaction propensity prev_tIP of amino acid triplets used in our previous study [7], and the interaction propensity tIP of amino acid triplets used in this study. The results shown in Table 3 were obtained by 5-fold cross validation with a window size of 15. The SVM models with the IP of the amino acid triplets (i.e., prev_tIP and tIP) were better than those with the IP of single amino acids (sIP). As a single feature, the new IP of amino acid triplets (tIP) showed the best performance. When the IP was used along with the RNA feature elements (R*_A_*, R*_C_*, R*_G_*, R*_U_*), performance always improved compared to the prediction with the IP only.

**Table 3:** The effect of IP and RNA features on prediction performance.

Features	Sn (%)	Sp (%)	Pr (%)	Ac (%)	NP (%)	Fm (%)	CC
sIP	41.3	70.9	13.2	68.1	56.1	52.2	0.08
prev_tIP	57.0	83.5	27.0	80.9	70.3	67.8	0.29
tIP	57.4	83.8	27.5	81.3	70.6	68.2	0.30
sIP + R	57.3	83.1	26.7	80.6	70.2	67.8	0.29
prev_tIP + R	61.5	85.1	30.7	82.8	73.3	71.4	0.34
tIP + R	61.4	85.2	30.8	82.9	73.3	71.4	0.35

The non-redundant dataset with a window size=15 was encoded into the feature vectors with different interaction propensities separately or with the RNA feature (R) in combination. The prediction performance was evaluated by 5-fold cross validation. sIP: the interaction propensity of single amino acids. prev_tIP: the previous version of the interaction propensity of amino acid triplets. tIP: the modified interaction propensity of amino acid triplets. Sn: sensitivity. Sp: specificity. Pr: precision rate. Ac: accuracy. NP: net prediction. Fm: F-measure. CC: correlation coefficient.

### Implementation and prediction results

We implemented a set of programs for extracting H bonds with various geometric criteria from PDB files, for encoding a feature vector for the data, and for running a SVM model with several different conditions. A program called HBPLUS (http://www.biochem.ucl.ac.uk/bsm/hbplus/home.html) is widely used to extract H bonds from PDB files, but it cannot deal with new PDB files that have atom names such as O2′ and OP1 in RNA. In our program for extracting H bonds, atoms O2′ and O3′ of RNA were included as potential donors of H bonds. Likewise, atoms OP1, OP2, O2′, O3′, O4′ and O5′ of RNA were included as potential acceptors of H-bonds.

To evaluate the effect of the window size on predicting RNA-binding amino acids, several datasets were constructed by applying the feature vector-based redundancy method with various window sizes to the PRI3149 dataset. Table 4 shows the prediction performance of the 5-fold cross validation on the SVM models trained by 8 different non-redundant datasets built from the PRI3149 dataset (see Table 1). All the results shown in Table 4 were obtained with the following parameter values: C=10, γ=1/#feature elements in the dataset, *w*+ (weight of positive class)=#negative feature vectors/#positive feature vectors, and *w-*(weight of negative class)=1. The prediction performance improved as the window size increased up to 15 in all performance measures. Therefore, the best prediction performance (an accuracy of 84.2%, an F-measure of 76.1%, and a correlation coefficient of 0.41) of 5-fold cross validation was achieved from the SVM model with a window size=15.

**Table 4:** The prediction performance with different window sizes.

Window size	Sn (%)	Sp (%)	Pr (%)	Ac (%)	NP (%)	Fm (%)	CC
1	47.3	73.6	16.1	71.1	60.4	57.6	0.14
3	66.5	84.8	30.9	82.7	74.1	72.6	0.36
5	64.9	84.5	30.9	82.6	74.7	73.4	0.36
7	65.6	84.9	31.7	83.0	75.2	74.0	0.37
9	66.8	84.9	32.2	83.2	75.9	74.8	0.38
11	66.8	85.4	32.8	83.6	76.1	74.9	0.39
13	67.1	85.8	33.7	84.0	76.5	75.3	0.40
15	68.4	85.9	34.1	84.2	77.1	76.1	0.41

The non-redundant dataset with various window sizes were constructed by applying the feature vector-based redundancy reduction method to the PRI3149 dataset. All 9 features were encoded in the feature vector. The prediction performance across varying window sizes was evaluated by 5-fold cross validation. Sn: sensitivity. Sp: specificity. Pr: precision rate. Ac: accuracy. NP: net prediction. Fm: F-measure. CC: correlation coefficient.

### Comparison with other methods

We compared our method to other machine learning methods for predicting RNA-binding amino acids in a protein sequence. BindN [2] uses a support vector machine with different amino acid features. RNABindR [3,4] predicts RNA-binding residues in a protein sequence using a Naïve Bayes classifier. These methods use different features to encode a feature vector and different machine learning algorithms to build a prediction model, but they performed the sequence similarity-based redundancy reduction method for their training datasets and did not consider the information of interacting partner sequences in predicting RNA-binding residues.

To objectively compare these methods we tested them on the PRI727 dataset and the PRI267 dataset, separately. Both the PRI727 and the PRI267 datasets were different from the PRI3149 dataset, which was used to train our SVM model. Table 5 shows the prediction performance of the methods with various options. RNABindR was run with three options: high sensitivity (sn), high specificity (sp) and optimal (opt). BindN was executed with two options: expected sensitivity of 80% (sn80) and expected specificity of 80% (sp80). In order to examine the effect of using the RNA feature on the prediction performance, we built two SVM models that used different features. ‘Our method 1’ in Table 5 represents an SVM model that used the RNA features as well as the 8 features (2 global features and 6 local features) of protein. ’Our method 2’ represents an SVM model that used the 8 protein features only. For both ‘Our method 1’ and ‘Our method 2’, the feature vector-based method was used to construct non-redundant training datasets from the PRI3149 dataset.

**Table 5:** Comparison of prediction methods on the PRI727 and PRI267 datasets.

Approach	Sensitivity (%)	Specificity (%)	Accuracy (%)	NP (%)	Fm (%)	CC
PRI727 dataset						

RNABindR (opt)	51.5	90.4	88.0	70.9	65.6	0.31
RNABindR (sp)	29.6	96.6	92.4	63.1	45.3	0.29
RNABindR (sn)	90.2	45.9	48.6	68.0	60.8	0.18
BindN (sn80)	88.4	51.0	53.4	69.7	64.7	0.19
BindN (sp80)	67.2	77.2	76.5	72.2	71.8	0.25
Our method 1	87.2	81.7	82.1	84.5	84.4	0.40
Our method 2	82.1	76.4	76.8	79.2	79.1	0.32

PRI267 dataset						

RNABindR (opt)	4.0	98.4	96.4	51.2	7.6	0.03
RNABindR (sp)	0.8	99.8	97.8	50.3	1.7	0.02
RNABindR (sn)	65.9	57.9	58.1	61.9	61.7	0.07
BindN (sn80)	80.2	53.5	54.0	66.8	64.2	0.10
BindN (sp80)	45.6	80.2	79.5	62.9	58.1	0.09
Our method 1	60.7	91.0	90.3	75.8	72.8	0.24
Our method 2	48.0	85.6	84.8	66.8	61.5	0.13

’Our method 1’ used all the 9 features (2 global features and 6 local features of protein and the RNA feature), whereas ’Our method 2’ used the 8 protein features (2 global features and 6 local features of protein) only. sn: high sensitivity option. sp: high specificity option. opt: optimal option. sn80: expected sensitivity of 80%. sp80: expected specificity of 80%. NP: net prediction. Fm: F-measure. CC: correlation coefficient.

**Figure 5:**
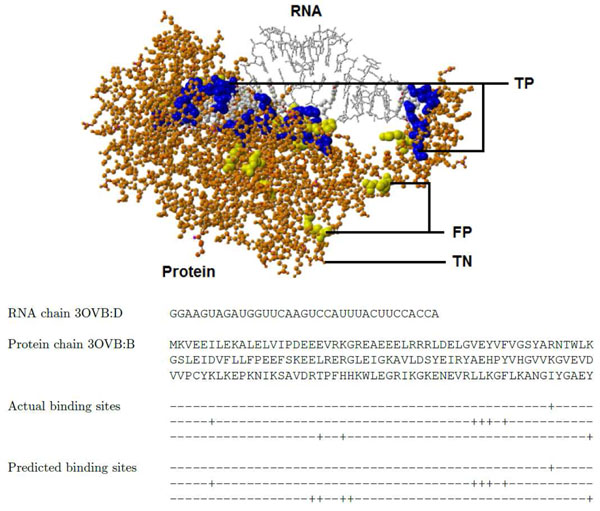
**Prediction of binding sites in protein chain B with RNA chain D of 3OVB**. 21 binding amino acids (blue balls, TP) and 408 non-binding amino acids (orange ball and sticks, TN) were predicted correctly. 12 non-binding amino acids were incorrectly predicted as binding (yellow balls, FP), and there was no binding amino acids that were incorrectly predicted as non-binding (no FN). In RNA protein-binding nucleotides are represented in dark gray balls and sticks, and non-binding nucleotides in gray wireframes. The ’+’ symbol below in the text line represents a binding amino acid while the ’-’ symbol represents a non-binding amino acid. Due to the limited space, the last 295 amino acids of protein chain B are not shown in the sequences. TP: true positives. TN: true negatives. FP: false positives. There are no false negatives (FN) in this example.

In both the PRI727 and the PRI267 datasets, our SVM model that used the RNA features as well as the protein features (Our method 1) had higher values for both sensitivity and specificity, but the other methods had either high sensitivity or specificity. As well as the high sensitivity and the high specificity, our SVM model (Our method 1) had the higher values for the net prediction, F-measure and the correlation coefficient than the other methods including our SVM model that used protein features only (Our method 2). Our SVM model that used protein features only (Our method 2) achieved the similar or better prediction performance than the existing methods. This result shows the feature vector-based method and the features are useful to construct a highly accurate prediction model in prediction of RNA-binding residues. Details of the prediction results are available in Additional Files 6 and 7. Figure 5 shows an example of prediction by the SVM model (Our method 1 in Table 5) for protein chain B with RNA chain D in a protein-RNA complex (PDB ID: 3OVB).

## Conclusions

Most learning approaches to predicting RNA-binding residues in a protein sequence construct a training dataset based on the sequence similarity. During the process of removing redundancy in sequence data a whole sequence is either taken or discarded for the training dataset. Similar sequences or even identical sequences often have very different binding sites when their binding partners change. However, much binding information is lost when a training data is constructed by the sequence similarity-based redundancy reduction method.

We developed a feature vector-based method for removing data redundancy. Our method constructed a larger training dataset of non-redundant data than the standard sequence similarity-based reduction method. Furthermore, the training dataset constructed by the feature vector-based method did not contain redundant data, whereas the dataset built by the sequence similarity-based method was likely to produce redundant data when a single sequence contains similar subsequences within the sequence.

Previous approaches to predicting RNA-binding residues in a protein sequence do not consider the interacting partner (i.e., RNA) of a protein. As a result, they always predict the same RNA-binding sites for a given protein sequence even if the protein binds to different RNA molecules. We took both protein and RNA sequences as input, and considered RNA feature to predict RNA-binding residues in the protein sequence. Our SVM model showed an accuracy of 84.2%, an F-measure of 76.1%, and a correlation coefficient of 0.41 with 5-fold cross validation on the 3,149 protein-RNA interacting pairs.

For a more rigorous evaluation we tested our SVM model on two new datasets, which were not used in training the model. In the new datasets, our SVM model showed an accuracy of 90.3%, an F-measure of 72.8% and a correlation coefficient of 0.24. Comparison of our SVM model with other methods on the same datasets demonstrated that ours is better than other methods.

In this study we identified effective features of protein and RNA (i.e., the interaction propensity of amino acid triplets and RNA features) for predicting RNA-binding residues and developed a new data redundancy method for constructing a training dataset. These will be useful in other studies of protein-RNA interactions.

## Competing interests

The authors declare that they have no competing interests.

## Authors' contributions

SC conceived the idea of removing data redundancy based on feature vectors, implemented it and prepared the first draft of the manuscript. KH supervised the work and rewrote the manuscript. All authors read and approved the final manuscript.

## Supplementary Material

Additional file 1 **The PRI3149 dataset.** 3,149 protein-RNA interacting pairs. Click here for file

Additional file 2 **The PRI727 dataset**. 727 protein-RNA interacting pairs. Click here for file

Additional file 3 **The PRI267 dataset**. 267 protein-RNA interacting pairs. Click here for file

Additional file 4 **The interaction propensity of the amino acid triplets** The interaction propensity of the amino acid triplets with 4 nucleotides, computed by equation (1) with the PRI3149 dataset Click here for file

Additional file 5 **The RNA feature of the 312 RNA sequences** The RNA feature computed for the 312 RNA sequences by equation (3) Click here for file

Additional file 6 **Prediction results with the PRI727 dataset** Prediction of RNA-binding amino acids in the PRI727 dataset Click here for file

Additional file 7 **Prediction results with the PRI267 dataset** Prediction of RNA-binding amino acids in the PRI267 dataset Click here for file
